# Retraction Note: M1 Macrophage-Derived Exosomal MicroRNA-326 Suppresses Hepatocellular Carcinoma Cell Progression Via Mediating NF-κB Signaling Pathway

**DOI:** 10.1186/s11671-022-03763-8

**Published:** 2022-12-13

**Authors:** Zhen-zi Bai, Hong-yan Li, Cheng-hua Li, Chuan-lun Sheng, Xiao-nan Zhao

**Affiliations:** Infectious Department, The Third Hospital of Jilin University, No. 126 Sendai Avenue, Changchun, 130033 Jilin China

**Retraction Note: Nanoscale Research Letters (2020) 15:221** 10.1186/s11671-020-03432-8

The Editors in Chief have retracted this article. After publication, concerns were raised about apparent partial overlaps within each image of Figure 1C, as well as unlikely appearance of the data presented in Figure 1f and the flow cytometry histograms in Figure 5a and e. In addition, liver cell lines used in the study (HL-7702, BEL-7404, SMMC-7721 and QGY-7703) have been previously reported as contaminated with HeLa cells and therefore not an appropriate model for this study. The authors did not respond to requests for further clarification and did not supply raw images or the ethics permit. The editors, therefore, have lost confidence in the integrity of the article’s findings. The authors did not respond to any correspondence from the editor about this retraction.

